# Effect of Intermittent Pneumatic Compression Device on Limb Edema in Immediate Postoperative Stroke Patients

**DOI:** 10.7759/cureus.110811

**Published:** 2026-06-14

**Authors:** Sakshi P Kavitake, Suraj Kanase

**Affiliations:** 1 Neurosciences, Krishna College of Physiotherapy, Krishna Vishwa Vidyapeeth (Deemed to Be University), Karad, IND

**Keywords:** intermittent pneumatic compression, limb edema, neurorehabilitation, physiotherapy, postoperative stroke

## Abstract

Introduction

Limb edema is a common complication in postoperative stroke patients, often caused by immobility and impaired lymphatic drainage. This swelling can negatively affect recovery. Intermittent pneumatic compression (IPC) devices, which enhance circulation by applying sequential pressure to the limbs, have shown promise in improving venous return and reducing edema in other patient groups. However, their effectiveness in managing limb edema in immediate postoperative stroke patients remains unclear. This study aims to assess the impact of IPC devices on reducing edema in this patient population.

Method

This quasi-experimental pre-post comparative study was conducted in the Neuro Intensive Care Unit of Krishna Vishwa Vidyapeeth, Karad. 30 immediate postoperative stroke patients with lower limb edema were included in the study and divided into two groups. Group A received IPC therapy along with conventional physiotherapy, while Group B received conventional physiotherapy alone. Both groups underwent treatment sessions for a duration of two weeks. Outcome measures were assessed pre and post intervention using limb girth measurements and the pitting edema scale. Statistical analysis was performed using paired and unpaired t-tests.

Result

Both groups demonstrated improvement following intervention; however, Group A showed significantly greater reduction in limb edema compared to Group B. Between-group analysis of the edema scale demonstrated statistically significant improvement in Group A (p = 0.0003). Limb girth measurements also showed significantly greater reduction in Group A compared to Group B (p = 0.0344).

Conclusion

IPC appears to be an effective adjunctive non-invasive intervention for managing post-operative lower limb edema in stroke patients, potentially supporting edema reduction and early recovery. However, these findings should be interpreted with caution given the small sample size and short intervention duration.

## Introduction

Stroke is a neurological condition resulting from an interruption of cerebral blood flow caused by either ischemic or hemorrhagic events, leading to neuronal injury and subsequent functional impairment [1]. Patients who undergo surgical intervention following stroke frequently require prolonged immobilization during the immediate post-operative period, predisposing them to several secondary complications that may adversely affect recovery and rehabilitation outcomes [2].

Lower limb edema is a common and clinically significant complication in postoperative stroke patients, particularly among individuals with hemiplegia or hemiparesis. Reduced muscle activity, impaired venous return, and compromised lymphatic drainage contribute to the accumulation of interstitial fluid within the affected limb [3,4]. Persistent edema may negatively affect mobility, delay rehabilitation, impair wound healing, and increase the risk of complications such as deep vein thrombosis (DVT), pressure ulcers, and venous congestion [5,6].

Various therapeutic approaches have been employed to manage postoperative limb edema, including limb elevation, compression garments, manual lymphatic drainage, and therapeutic exercise programs [7]. Although these interventions may provide symptomatic relief, their effectiveness can be limited during the acute post-operative phase, particularly in critically ill patients with reduced mobility and impaired participation in rehabilitation activities [8].

Intermittent pneumatic compression (IPC) is a non-invasive therapeutic modality that utilizes inflatable cuffs or sleeves to deliver cyclical external pressure to the limbs. This sequential compression-decompression mechanism mimics the physiological muscle pump action, thereby enhancing venous return, reducing venous stasis, promoting lymphatic drainage, and improving peripheral circulation [6,7]. In addition to its established role in the prevention of DVT, IPC has demonstrated beneficial effects in reducing limb swelling, improving tissue oxygenation, and facilitating fluid resorption in various clinical populations [9,10].

Recent evidence suggests that IPC may contribute to improved circulatory dynamics and reduction of edema-related complications in patients with neurological impairments [11]. Furthermore, enhanced circulation and reduced fluid accumulation may facilitate participation in rehabilitation programs and support functional recovery [12]. Despite these potential benefits, evidence regarding the effectiveness of IPC in immediate post-operative stroke patients remains limited, particularly in individuals admitted to neuro-intensive care settings. Therefore, the present study aimed to evaluate the effect of IPC on lower limb edema in immediate post-operative stroke patients.

## Materials and methods

Study design and setting

This quasi-experimental pre-post comparative study was conducted among immediate postoperative stroke patients presenting with lower limb edema. Participants were assigned to either the experimental group or the control group using an alternate allocation method, in which consecutive eligible participants were allocated alternately between the two groups. The study was carried out in the Neuro Intensive Care Unit at Krishna College of Physiotherapy. Ethical approval was obtained from the Institutional Ethics Committee on November 5, 2025 (KVV/IEC/10/2025), following which patient recruitment commenced in November 2025 and continued until March 2026, allowing all enrolled participants to complete the two-week intervention protocol within the six-month study duration. The sample size was calculated using the formula: ((Z₁−α/2 + Zp)²)/(P₁−P₂)². The sample size was determined using a significance level (α) of 0.05 and a statistical power of 80% (β = 0.20). Based on an anticipated moderate effect size derived from previous literature evaluating edema reduction interventions in neurological patients, the minimum required sample size was calculated. To compensate for potential dropouts and ensure adequate statistical power, a total of 30 participants were included in the study. Based on the calculation, a total of 30 participants were included in the study.

Participants

A total of 30 immediate postoperative stroke patients with lower limb edema who fulfilled the inclusion criteria were enrolled in the study. All included participants were cases of acute cerebrovascular events who had undergone neurosurgical intervention, including decompressive craniectomy and evacuation of intracerebral hematoma, based on clinical and radiological indications. Postoperatively, patients were managed in the neurosurgical intensive care unit with standard care and were referred for physiotherapy once clinically stable and eligible for participation.

Participants were allocated into two groups using an alternate allocation method. Consecutive eligible participants were assigned alternately to Group A, which received IPC in combination with conventional physiotherapy, and Group B, which received conventional physiotherapy alone. Written informed consent was obtained from the caregivers of all participants prior to study enrollment.

Inclusion and exclusion criteria 

Patients aged 40-65 years; immediate postoperative stroke patients with lower limb edema; patients receiving mechanical ventilatory support with stable hemodynamic status, defined as stable heart rate and blood pressure without the need for escalation of vasopressor therapy; Glasgow Coma Scale score between 3 and 8; both male and female participants were included. Participants with uncontrolled hypertension, severe cardiac disease, peripheral vascular disease, DVT, severe skin infection or open wounds over the treatment area were excluded from the study.

Data source and tools

The control group received conventional physiotherapy, which included positioning, passive range of motion (ROM) exercises, bed mobility training, breathing exercises, and limb elevation techniques. The experimental group received IPC therapy along with conventional physiotherapy. Both groups underwent supervised treatment sessions lasting 35-40 minutes per session, administered once daily, six days per week, over a two-week intervention period. Outcome measures included limb girth measurement using a non-elastic measuring tape at standardized anatomical landmarks (mid-thigh measured at the midpoint between the anterior superior iliac spine and the superior border of the patella, and mid-calf measured at the widest part of the gastrocnemius muscle), along with the Edema Grading Scale to assess the severity of lower limb edema. All measurements were performed by a single trained assessor to ensure intra-rater consistency. Data were collected pre- and post-intervention, statistically analyzed, and presented in a summarized form upon completion of the study.

The intervention was conducted over a period of two weeks, with participants allocated into two groups: an experimental group (Group A) receiving IPC along with routine physiotherapy, and a control group (Group B) receiving routine physiotherapy alone. Each treatment session lasted approximately 35-40 minutes and was performed under professional supervision.

During Week 1, both groups began treatment with positioning and limb elevation techniques to reduce dependent edema and improve circulation. Group A received IPC therapy for 30 minutes per session, with calf pressure maintained at 40-50 mmHg and thigh pressure at 50-60 mmHg. The inflation-deflation cycle consisted of 11-12 seconds of inflation followed by 50-60 seconds of deflation [13-16]. These parameters and treatment duration were selected based on previously published studies and established clinical protocols for edema management and neurorehabilitation. Group B received gentle limb massage as part of edema management. Both groups additionally performed passive and active-assisted ROM exercises for all major joints (10 repetitions each) along with ankle pump exercises (15 repetitions × 2 sets). Bed mobility exercises including rolling and bridging were gradually introduced and progressed during the week. Repetitions of ROM exercises and ankle pumps were increased according to patient tolerance.

In Week 2, progression of the treatment protocol was introduced by increasing exercise repetitions and incorporating functional mobility activities. Group A continued IPC therapy for 30 minutes along with ROM exercises increased to 15 repetitions, ankle pumps (20 repetitions), and bridging exercises progressed up to 15 repetitions. Assisted bed-to-chair transfer training and transfer practice were also included to improve functional mobility. Group B continued routine physiotherapy consisting of limb massage with compression techniques, ROM exercises, ankle pump exercises, bridging activities, and assisted transfer training with similar progression in repetitions and functional activities.

All treatment sessions in both groups concluded with relaxation positioning and breathing exercises. This structured progression ensured gradual improvement in circulation, reduction of limb edema, maintenance of joint mobility, and enhancement of functional independence, with the primary difference being the addition of IPC therapy in the experimental group.

Statistical analysis 

All data were entered into a Microsoft Excel spreadsheet (Microsoft Corp., USA) and statistically analyzed using GraphPad InStat software (version 3.06, trial version; GraphPad Software, Inc., USA). Data distribution was assessed for normality prior to analysis to determine the appropriateness of parametric testing. Descriptive statistics, including mean and SD, were calculated for all outcome measures. A paired t-test was used to assess within-group differences between pre-intervention and post-intervention values, while an unpaired t-test was used to compare differences between the experimental and control groups. The outcome measures included limb girth measurement using a measuring tape and the Edema Grading Scale. A p-value of less than 0.05 was considered statistically significant.

## Results

Table 1 presents the gender distribution of the study participants, including frequency and percentage of males and females.

**Table 1:** Gender distribution of the study participants

Gender	Frequency (n)	Percentage (%)
Male	20	66.6
Female	10	33.3
Total	30	100

The comparison of pre-test and post-test limb edema scores between Group A (IPC with routine physiotherapy) and Group B (conventional physiotherapy) is presented in Table 2. At baseline, there was no statistically significant difference between the two groups. However, postintervention analysis demonstrated a marked reduction in edema in Group A compared with Group B (p = 0.0003). Furthermore, the mean improvement observed in Group A was greater than that of Group B, indicating superior effectiveness of IPC in reducing limb edema.

**Table 2:** Comparison of scores between Group A and Group B IPC: Intermittent pneumatic compression

Parameter	Group A (IPC)	Group B (Conventional physiotherapy)	Mean difference (A-B)	t-value	p-value	Significance	
Pre-test mean ± SD	3.6 ± 1.4	3.46 ± 0.516	0.14	0.363	0.7206	Not significant	
Post-test mean ± SD	0.507 ± 0.507	0.51 ± 0.703	-0.003	-0.013	0.9894	Not significant	
Mean improvement (pre-post)	2.2 ± 0.56	1.4 ± 0.5	-0.8	4.099	0.0003	Extremely significant	

The comparison of pre-test and post-test limb girth measurements between Group A (IPC) and Group B (conventional physiotherapy) is presented in Table 3. Postintervention analysis showed a significant reduction in limb girth in both groups, with greater improvement observed in Group A compared to Group B (p = 0.0344), indicating better effectiveness of IPC.

**Table 3:** Comparison of limb girth measurement scores between Group A and Group B IPC: Intermittent pneumatic compression

Parameter	Group A (IPC)	Group B (Conventional physiotherapy	Mean difference (A-B)	t-value	p-value	Significance
Pre-test mean ± SD	35.4 ± 30.9	35.1 ± 31.9	0.3	0.026	0.9793	Not Significant
Post-test mean ± SD	4.53 ± 4.6	4.37 ± 4.02	0.16	0.101	0.9199	Not Significant
Mean improvement (pre-post)	4.46 ± 3.2	1.76 ± 1.32	-1.297	2.224	0.0344	Significant

## Discussion

This study evaluated the effectiveness of IPC in reducing limb edema in immediate postoperative stroke patients. A total of 30 participants (20 males and 10 females), aged between 40 and 65 years, were included and equally allocated into two groups (n=15 per group). Group A received IPC along with conventional physiotherapy, while Group B received conventional physiotherapy alone. The results demonstrated a significantly greater reduction in limb edema in the IPC group compared to the control group, indicating the superior effectiveness of IPC in early postoperative management.

IPC is widely used in clinical practice for the management of edema and prevention of DVT. It works by applying intermittent cyclical pressure to the limbs, thereby enhancing venous return, reducing venous stasis, and facilitating lymphatic drainage [17]. These physiological effects are particularly important in postoperative stroke patients, who often experience reduced mobility and impaired circulatory dynamics [18]. In the present study, the addition of limb positioning and routine physiotherapy further supported venous return and contributed to improved outcomes in the experimental group. In contrast, the control group received conventional physiotherapy, which included limb movements, positioning, and manual edema management techniques. Although these interventions help maintain joint mobility and promote circulation to some extent, they do not provide the same consistent external mechanical pumping effect as IPC. This may explain the comparatively lesser reduction in edema observed in Group B.

Poststroke edema is a multifactorial condition resulting from immobility, neurological impairment, and altered vascular and lymphatic function. Following stroke and surgery, reduced muscle activity limits the natural muscle pump mechanism, leading to fluid accumulation in dependent limbs [19]. If not managed effectively, edema can contribute to pain, restricted ROM, delayed functional recovery, and increased risk of complications such as pressure ulcers and thromboembolic events [20]. IPC acts as a mechanical substitute for the impaired muscle pump by cyclically compressing and decompressing the limb. This promotes distal-to-proximal fluid movement, reduces interstitial fluid accumulation, and improves overall tissue perfusion [21]. Enhanced circulation also supports oxygen and nutrient delivery to tissues, which may accelerate healing and improve rehabilitation outcomes in postoperative stroke patients [22]. Previous studies have also reported that early application of IPC in postoperative and immobilized patients significantly reduces limb volume and improves comfort [23]. In addition, IPC has been shown to reduce the risk of secondary complications such as DVT and venous congestion. However, its effectiveness may vary depending on patient-specific factors such as severity of neurological impairment, level of mobility, and tolerance to compression therapy [24]. Despite its benefits, IPC should not be considered a standalone intervention. A comprehensive rehabilitation approach that includes physiotherapy, mobilization strategies, and functional training remains essential for optimal recovery [25]. Patient compliance and appropriate timing of intervention also play important roles in determining outcomes [26].

Overall, the findings of this study support the use of IPC as an effective adjunct therapy for reducing limb edema in immediate post-operative stroke patients. Its early application in rehabilitation may enhance recovery, improve functional outcomes, and reduce complication risk. However, further large-scale randomized controlled trials are recommended to establish standardized protocols and long-term effectiveness of IPC in this patient population.

## Conclusions

IPC may be a beneficial adjunctive intervention for reducing lower limb edema in immediate postoperative stroke patients. In this study, patients who received IPC along with conventional physiotherapy demonstrated greater improvement in edema reduction and limb girth measurements compared to those receiving conventional physiotherapy alone. These findings indicate a potential role of IPC in enhancing venous return, promoting lymphatic drainage, and improving peripheral circulation, which may support early postoperative recovery. Early management of limb edema remains an important component of stroke rehabilitation to help minimize complications and support functional outcomes.

However, the results should be interpreted with caution due to the small sample size, short intervention duration of two weeks, and absence of long-term follow-up. Therefore, the findings cannot be generalized to a wider population without further evidence.

This study has certain limitations that should be acknowledged. The sample size was relatively small (n=30) and drawn from a single center, which may limit the generalizability of the findings. The intervention period was short (two weeks), and no long-term follow-up was conducted to assess the persistence of the observed effects. Additionally, the study included only patients with a Glasgow Coma Scale score of 3-8, which may restrict applicability to other neurological severity levels. Therefore, IPC may serve as a beneficial non-invasive adjunctive intervention for managing post-operative limb edema in stroke patients, pending confirmation from larger randomized controlled trials.
